# The effects of uterine size with or without abdominal obesity on spinal block level and vasopressor requirement in elective cesarean section: a prospective observational study

**DOI:** 10.3906/sag-1804-167

**Published:** 2019-02-11

**Authors:** İlkben GÜNÜŞEN, Asuman SARGIN, Ali AKDEMİR, Ahmet Mete ERGENOĞLU

**Affiliations:** 1 Department of Anesthesiology and Reanimation, Faculty of Medicine, Ege University, Izmir Turkey; 2 Department of Obstetrics and Gynecology, Faculty of Medicine, Ege University, Izmir Turkey

**Keywords:** Spinal anesthesia, sensory level, hypotension, fundal height, abdominal obesity

## Abstract

**Background/aim:**

Hypotension is a serious complication caused by spinal anesthesia that places both the mother and fetus at increased risk. We aimed to investigate the effects of uterine size with or without abdominal obesity on sensory block level of pregnant women receiving spinal anesthesia.

**Materials and methods:**

This study included 125 term parturients who underwent cesarean section. Motor and sensory block characteristics, the distance between the symphysis pubis and the fundus (SPF), the distance between the symphysis pubis and the xiphoid (SPX), newborn and placental weights, adverse effects, and doses of ephedrine were recorded.

**Results:**

Sensory block level and ephedrine dose were significantly correlated with the SPX and the combined newborn and placenta weights (P < 0.05). The incidence of hypotension was related to the SPX and the combined newborn and placenta weight (P < 0.05). There was no correlation between the SPF and sensory block level or ephedrine dose. The sensory block level was higher for patients who had greater SPX values and higher combined newborn and placenta weights. The incidence of hypotension and the ephedrine dose were also higher in these subjects.

**Conclusion:**

SPX values and combined newborn and placenta weights are more predictive of sensory block level than SPF values in parturients receiving spinal anesthesia.

## 1. Introduction

Spinal anesthesia is a safe, simple, rapid, effective, and easy-to-apply anesthetic technique for cesarean sections. Despite the advantages of spinal anesthesia, hypotension is a common complication with an incidence of 70%–85% in parturients (1–4). Many factors including the characteristics of the injected solution, patient position, age, height, weight, pregnancy, intraabdominal pressure (IAP), length of the vertebral column, and lumbosacral cerebrospinal fluid (CSF) volume determine the intrathecal spread of local anesthetics. In obstetric patients, physiological changes due to pregnancy, including changes in spinal curvature, venous pooling secondary to progesterone-induced decreases in vascular tone and aortocaval compression by the gravid uterus, contribute to hypotension during cesarean section under regional anesthesia (1–6). 

Many recent studies have addressed the effects of uterine size, abdominal girth, and IAP on sensory block level, ephedrine dose, and the incidence of hypotension in pregnant women. Large abdominal girth, big uterus, and increased IAP may be significant factors in greater cephalad spread of local anesthetics. The mechanisms explained in these studies are that the increased IAP secondary to the compression of the inferior vena cava due to the growing uterus leads to a reduction in CSF in the spinal canal (1,7–14). 

We hypothesized that there may be differences in the sensory block level and ephedrine requirements among pregnant women based on uterine size and the presence of abdominal obesity. The primary purpose of this study was to determine whether there is a relationship between uterine size and sensory block level and ephedrine dose in parturients receiving spinal anesthesia. Since Katulanda et al. (15) reported that the distance between the lower border of the xiphisternum and the center of the umbilicus could be used as an anthropometric measure to define abdominal obesity, we considered SPX to be a marker of both abdominal obesity and uterine size that contains abdominal obesity, easily identified anatomical landmarks in pregnant women. Therefore, we aimed to investigate the correlations related to SPX and sensory block levels and ephedrine requirements.

## 2. Materials and methods

We conducted this prospective, observational study between 2012 and 2017 in patients who were between 37 and 40 weeks of gestation. The study was approved by an ethics committee (Ege University Clinical Research Ethics Committee of the Faculty of Medicine, 2011, 1600/639), and written informed consent was obtained from each patient. The 125 pregnant women with an American Society of Anesthesiologists (ASA) physical status II underwent elective cesarean section under spinal anesthesia. Parturients aged between 20 and 40 years old were having uncomplicated, singleton, and term pregnancy. We excluded patients who had contraindications to spinal anesthesia, allergic reactions to local anesthetics, preeclampsia, cardiac diseases, diabetes mellitus, gestational age <37 weeks, morbid obesity (BMI > 35), multiple gestations, height <150 cm or >175 cm, placental or fetal abnormalities, and poly- or oligohydramnios.

Chung et al. (8) used symphysis-fundal height to assess the size of the uterus. Deeluea et al. (16) reported that birth weight was directly correlated with fundal height, as the uterus expanded to accommodate the fetus, placenta, and amniotic fluid. Studies have generally reported that fetal weight affects peak sensory block level; however, the weights of the amniotic fluid and placenta have been overlooked. In our study, preoperative evaluations included obstetric ultrasound examinations to assess the volume of the amniotic fluid. Amniotic fluid index (AFI) was measured by dividing the uterus into four imaginary quadrants; patients with AFI values ranging from the 50th to 95th percentiles were included in the study. Based on previous studies (8,16), we used two methods to determine uterine size: first, the combined newborn and placenta weights were recorded in cases where AFI ranged from the 50th to 95th percentiles and second, the distance between the symphysis pubis and the fundus of the uterus (SPF) was measured as described by Chung et al. (8). The SPF (from the mother’s pubic bone to the fundus of the uterus) was conventionally measured by an obstetrician with the patient in a supine position. The SPX (from the mother’s pubic bone to xiphoid process) was also measured in a similar manner before the operation.

Prior to transfer to the operating room, baseline vital parameters including systolic blood pressure (SBP) and heart rate (HR) were measured at rest. A 16-gauge cannula was inserted into a forearm vein, and 50 mg of ranitidine and 10 mg of metoclopramide were administered intravenously to parturient one hour before surgery. All the patients received standard fluid therapy with Ringer’s lactate solution 15 mL kg−1 over a period of 20 min prior to the surgery. Physiological monitoring, including automated noninvasive blood pressure, electrocardiogram, HR, and pulse oximetry assessments, was performed in the operating room. Spinal anesthesia was administered using a 26-gauge Atraucan spinal needle (B. Braun, Melsungen, Germany) through a midline approach at the L3–4 interspace by one anesthetist in sitting position. A combination of 2 mL of 0.5% hyperbaric bupivacaine (Marcain Heavy, AstraZeneca, England) and 25 mcg (0.5 mL) of fentanyl (Fentanyl-Janssen, Janssen-Cilac) at room temperature was injected intrathecally over 10 s without barbotage while the patient was in this position. The patient was then placed in a supine position with a 15° left lateral tilt. Surgery proceeded if the upper sensory block level, as assessed by the loss of sensation to a pinprick, was ≥T5.

Maternal SBP and HR were recorded every min for the first 10 min after intrathecal injection, at 2-min intervals for the next 10 min and at 5-min intervals thereafter. Hypotension was defined as SBP ˂ 90 mmHg or a reduction of 20% or more than the baseline. If hypotension occurred, 5 mg of intravenous ephedrine was administered as a bolus and repeated every 2 min as necessary. Bradycardia was defined as HR < 50 beats per minute and was treated with 0.5 mg of intravenous atropine. 

Demographic characteristics, obstetric data, duration of surgery (from skin incision to closure), time from skin incision to delivery of the newborn, and newborn and placental weights were recorded. The lowest SBP and HR within 30 min of the intrathecal injection and the incidence of hypotension and adverse effects such as nausea and vomiting were noted. The maximum sensory block level, time taken to attain the maximum sensory block level, time to a two-segment regression from the maximum block height, time taken for the block to recede to the T10 level, degree of motor block and duration of sensory and motor blocks were recorded. The upper sensory levels were assessed in both midclavicular lines by the loss of a pinprick sensation using a 25-gauge Whitacre needle at 20 min after the intrathecal injection. The sensory block level at 15 min was defined as the level of the maximum sensory block. The motor block was assessed based on a modified Bromage scale (0, no paralysis, able to flex hips/knees/ankles; 1, able to move knees, unable to raise extended legs; 2, able to flex ankles, unable to flex knees; 3, unable to move any part of the lower limb). The duration of sensory or motor block was defined as the time from the intrathecal injection to T10 regression or to the point at which the Bromage score returned to zero. The incidence of side effects such as nausea, vomiting, and hypotension were noted; the total dose of ephedrine and atropine administered was also recorded. Then, 50 mcg of intravenous fentanyl was administered for any intraoperative discomfort after the delivery of the baby. Nausea and vomiting were intravenously treated with 4 mg of ondansetron if there was no hypotension. 

### 2.1. Statistical analysis

The study was designed to test three predictors of the spread of spinal anesthesia including the SPF, SPX, and combined newborn and placenta weight. The effect of these predictors on the maximum sensory block level, ephedrine dose, and incidence of hypotension were determined. The sensory block level was recorded as 2, 3, 4, and 5 for T2, T3, T4, and T5 dermatomal levels, respectively. Hypotension was coded as “1” if present and “0” if absent. As no information was available regarding the expected differences in SPX and SPF measurements, the required sample size was calculated based on our pilot study. A minimum sample size of 112 was needed to detect an anticipated effect size of 0.3 for the regression equation, at a power level of 0.95 (β = 0.95) and a probability level of 0.05 (α = 0.05). 

Statistical analyses were performed using the SPSS statistical package (SPSS for Windows, release 22.0). The results were presented as the mean ± standard deviation (SD), median (range), or n (%), as appropriate. Spearman’s rho test was used for correlation analyses between the SPF and SPX and the maximum sensory block level and ephedrine dose and between the combined newborn and placenta weights and the maximum sensory block level and ephedrine dose. Correlations between sensory block level and ephedrine dose and the SPF, SPX, and combined newborn and placenta weights were determined using multiple regression analyses. Correlation analyses were performed among these three predictors to identify those that predicted a sensory level of T5**.** The relationships between hypotension and the SPF, SPX, and combined newborn and placenta weights were tested with Mann–Whitney U and Wilcoxon W tests. A P-value of ˂0.05 was considered statistically significant. 

## 3. Results

One hundred and thirty-two patients were initially enrolled but seven patients were excluded from the study due to inadequate spinal block for surgical anesthesia (a sensory block level of T5 was not achieved) or failed spinal anesthesia. Maternal and newborn characteristics are shown in Table 1; details of surgeries and spinal anesthesia are presented in Table 2.

**Table 1:** Maternal and newborn characteristics. Values are shown as median (range) or means (SD).

Age (years)	31.55 ± 4.48
Weight (kg)	78.13 ± 9.17
Height (cm)	162.94 ± 6.16
BMI (kg m−2)	29.47 ± 3.16
Gestational age (weeks)	38 (37–40)
Parity	1 (0–3)
Newborn height (cm)	50.04 ± 2.21
Newborn weight (g)	3290.51 ± 366.23
Placenta weight (g)	583.16 ± 101.16
Combined newborn and placenta weight (g)	3873.67 ± 430.25
SPF (cm)	32.8 ± 4.05
SPX (cm)	41.6 ± 3.73
Baseline SBP (mmHg)	128.28 ± 9.96
Baseline HR (beats min−1)	92.64 ± 10.69
Lowest SBP (mmHg)	94.57 ± 14.49
Lowest HR (beats min−1)	73.11 ± 8.5

BMI: body mass index, SBP: systolic blood pressure, HR: heart rate, SPF: symphysis pubis–fundus distance, SPX: symphysis pubis–xiphoid distance.

**Table 2:** Surgical data and motor and sensory block characteristics. Values are shown as median (range) or mean ± SD.

Duration of surgery (min)	48.12 ± 9.91
Induction–delivery time (min)	12.33 ± 4.12
Motor block	
Maximum motor block score (Bromage)	3 (1–3)
Time to maximum motor block (min)	5.9 ± 3.92
Time for recovery to Bromage score of 0	151.22 ± 46.23
Sensory block	
Maximum sensory block level	T4 (2–5)
Time to maximum sensory block (min)	4.72 ± 2.5
Time to two-segment regression (min)	96.55 ± 36.23
Time to recede to T10 (min)	142.55 ± 43.09

The SPX and the combined newborn and placenta weights were negatively correlated with the maximum sensory block level (P = 0.001, P = 0.025, respectively). This relationship was inversely correlated because we defined T2, T3, T4, and T5 dermatomal levels as sensory levels 2, 3, 4, and 5, respectively. There was no significant correlation between the SPF and the maximum sensory block level. The dose of ephedrine was positively correlated to the SPX and the combined newborn and placenta weights; however, it was not correlated to the SPF (Table 3). 

**Table 3:** Correlations for SPF, SPX, and the combined newborn and placenta weights

	Maximum sensoryblock level		Ephedrinedose	
	r	p	r	p
SPF	0.077	0.396	0.044	0.624
SPX	−0.297**	0.001	0.267**	0.003
Combined newborn and placenta weights	−0.2*	0.025	0.303**	0.001
BMI	−0.343**	<0.0001	0.054	0.548

SPF: symphysis pubis–fundus distance, SPX: symphysis pubis–xiphoid distance, r: correlation coefficient. Spearman’s rho test was used to assess correlations between the maximum sensory block level and ephedrine dose and SPF, SPX, and the combined newborn and placental weights. *Correlation is significant at the 0.05 level (2-tailed) ** Correlation is significant at the 0.01 level (2-tailed).

There was a statistically significant relationship between the incidence of hypotension and the SPX and the combined newborn and placenta weights, although hypotension incidence was unrelated to the SPF (Table 4). Hypotension occurred in 65 of 125 cases (52%) and was accompanied by nausea and vomiting in 23 cases (35.4%). A mean ephedrine dose of 10.15 ± 6.31 mg was used in these patients; the mean dose administered for all patients was 5.28 ± 6.81 mg. Four patients required atropine for bradycardia (Table 5). The median maximum sensory block level was 4 (2–5) in hypotensive patients and 4 (3–5) in the other subjects. 

**Table 4:** The relationships among hypotension and SPF, SPX, and the combined newborn and placenta weights.

	SPF	SPX	Combined newbornand placenta weights
Hypotension	0.54	0.046*	0.042*

SPF: symphysis pubis–fundus distance, SPX: symphysis pubis–xiphoid distance. Mann–Whitney U and Wilcoxon W tests were used to compare hypotension and other outcomes. *P < 0.05.

**Table 5:** Side effects and ephedrine dose. Values are shown as the number (proportion) or mean ± SD.

Side effects	69 (55.2%)
Hypotension (n, %)	65 (52%)
Nausea and/or vomiting (n, %)	23 (18.4%)
Bradycardia (n, %)	4 (3.2 %)
Number of patients treated with ephedrine (n, %)	65 (52%)
Mean ephedrine dose in all patients (mg)	5.28 ± 6.81
Mean ephedrine dose in patients who were treated with ephedrine (mg)	10.15 ± 6.31

Correlation analyses showed an inverse relationship between BMI and maximum sensory block level (P ˂ 0.0001) (Table 3). However, we could not determine an association between BMI and the incidence of hypotension or the dose of ephedrine. Results of the regression analyses for the SPX and the combined newborn and placenta weights are shown in Table 6.

**Table 6:** Regression analyses for SPX and the combined newborn and placental weights.

	Sensory block level				Ephedrine dose			
	r	p	b	p	r	p	b	P
SPX	−0.297	0.001*	−1.420	0.011	0.267	0.003*	0.110	0.028
Combined newborn and placenta weights	−0.2	0.025*	−72.424	0.26	0.303	0.001*	17.599	0.003

SPX: symphysis pubis–xiphoid distance, r: correlation coefficient, b: coefficient, *P < 0.05.

**Figure 1:**
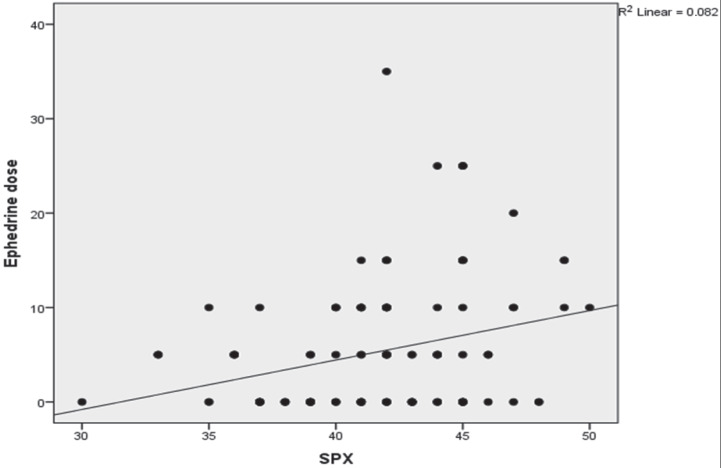
The relationship between SPX and administered dose of ephedrine.

**Figure 2:**
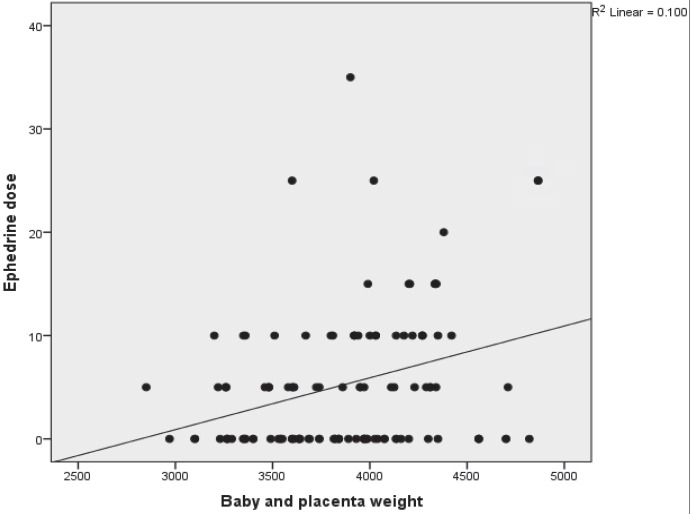
The relationship between the combined newborn and placenta weights and dose of ephedrine.

Multiple regression analyses revealed that these 2 general characteristics of a patient, i.e. the SPX and the combined newborn and placental weights, had a high predictive value for the spread of the sensory block and for the ephedrine requirement after spinal anesthesia (Figures 1 and 2). 

## 4. Discussion

We found that SPF measurements were unrelated to the maximum sensory block level, incidence of hypotension, and ephedrine dose. However, higher sensory block levels were seen in pregnant women who had longer SPX measurements and higher combined newborn and placenta weights. The incidence of hypotension and the ephedrine requirements were greater in these women.

 Fundal height, because of its direct relation to fetal and uterine size, was investigated to predict sensory block level and ephedrine dose in term parturients who underwent elective cesarean sections under spinal anesthesia (8). However, fundal height is generally used as a proxy for estimating the gestational age of a pregnancy and, more commonly, as an antenatal screening for abnormalities in fetal growth. Additionally, several factors, including BMI, parity, bladder volume, ethnicity, and difficulties in determining anatomical landmarks that result from individual errors, are known to greatly influence fundal height values. Fetal size and fundal height measurements vary significantly with different individual BMI values even within the normal BMI range; in addition, different combinations of height and weight lead to the same BMI value. The standard technique for SPF measurements involves the patient in a supine position on a firm surface with an empty bladder. However, measurements of fundal height may be incorrect in this position in pregnant women at term due to the displacement of the fundus toward the diaphragm in the last trimester as well as difficulties in determining anatomical landmarks (17–23). Chung et al. (8) found that the SPF was not significantly correlated with the maximum sensory block level similar to our study but was correlated with the dose of ephedrine administered for hypotension. A significant correlation was found between the administered ephedrine dose and the weight of the fetus. 

Many studies have shown that the relation between BMI and the percentage of body fat differs across ethnic groups and the pattern of obesity differs considerably from nation to nation (24–26). Asians generally have a higher percentage of body fat than white people of the same age, sex, and BMI (27). Therefore, apart from BMI, central adiposity may be considered a more accurate predictor of sensory block level after spinal anesthesia. Central or abdominal obesity during pregnancy may be more crucial than BMI in its effects on the cephalad spread of spinal anesthesia due to raised IAP and aortocaval compression (6–8,10,12,13). Abdominal obesity may be a more important factor than fundal height and BMI in terms of sensory block level, and BMI may not be a good method to measure abdominal adiposity (6,12–14,25). Fundus measurements may be affected by BMI and can lead to errors in diagnosing patients with abdominal obesity (17,18,28,29). Several anthropometric parameters are used for the identification of abdominal obesity, such as waist circumference, waist-to-hip ratio, and sagittal abdominal diameter (SAD) (15). An elevated waist circumference defines central obesity, and increased waist circumference and SAD are associated with an increase in IAP (29). In recent studies, the effect of abdominal circumference on the intrathecal spread of local anesthetics during spinal anesthesia was investigated, and larger abdominal girth was correlated with the level of spinal anesthesia (6,10,12,13). Wei et al. (6) showed that abdominal girth and the length of the vertebral column have significant predictive value in determining the cephalad spread of spinal anesthesia using hyperbaric bupivacaine in parturients; specifically, pregnant women with larger abdominal girth values and shorter vertebral column lengths often showed higher cephalad spread of spinal anesthesia. Zhou et al. (12) also demonstrated that patients with larger abdominal girth values and shorter vertebral column lengths have a higher cephalad spread. However, Kuok et al. (30) found no significant correlation between abdominal circumference and the maximum sensory block level, the incidence of hypotension, ephedrine dosage after spinal anesthesia; this finding may be due to the small sample size. In addition, Malbrain et al. (31) reported that there was a weak correlation between abdominal perimeter and IAP and that abdominal perimeter could not be used as a clinical estimate for IAP. Chun et al. (10) also found that IAP did not correlate with SAD. Although most of the studies have assessed abdominal obesity by measuring the abdominal girth in parturients (6,12–14), Katulanda et al. (15) suggested that the distance between the lower border of the xiphisternum and the center of the umbilicus could be used as an anthropometric measure to define abdominal obesity. Therefore, we decided to include the SPX as well as the SPF in our study. We assumed that the SPX, which reflects both abdominal obesity and uterine size, may be more predictive of the maximum sensory block than the abdominal circumference or diameter is. In this study, we found correlations among the SPX and the maximum of spinal block level and ephedrine dose, although SPF measurements were unrelated. Patients with longer SPX values had a higher sensory block level and a higher dose of ephedrine. 

Studies on patient characteristics such as body weight, height, and BMI show controversial spinal level and hypotension results (7,10,11,13,24,32). Norris et al. (24) found that weight, height, BMI, and vertebral column length were not correlated with the spread of the sensory block. In some studies, pregestational weight, rather than the weight gained during pregnancy, was found to be more important in determining the sensory block level (1,7,33). A pregestational BMI ≥ 25 kg m-2 is a risk factor for hypotension after spinal anesthesia in patients undergoing a cesarean section (1). Ekelof et al. (33) and Ozkan et al. (7) reported that cephalad spread is not related to the degree of weight gain during pregnancy. We found a significant correlation between BMI and the maximum sensory block level; however, the incidence of hypotension and ephedrine dose were unrelated to BMI. These findings may be because patients in the group without hypotension were taller than those in the group that developed hypotension (mean height 164.22 ± 6.52 cm and 161.75±5.6 cm, respectively). Thus, the median maximum sensory block level was T4 (2–5) in the hypotensive patients and T4 (3–5) in the other subjects. 

Chung et al. (8) found that the incidence of hypotension was 53.8%, similar to our study; the incidence of nausea and vomiting was 32.7%. The dose of ephedrine administered after spinal anesthesia was 7.5 ± 9.8 mg. In the present study, a variable dose of the local anesthetic was used to adjust for variations in height and weight. 

There are some limitations to our study. First, we only measured SPX in a supine position. The distance between the xiphisternum and center of the umbilicus measured in a standing position was studied previously as an indicator of abdominal obesity by Katulanda et al. (15). Second, we did not study the relation between SPX values and the length of vertebral column or abdominal girth. 

In conclusion, the maximum sensory block level, ephedrine dose, and hypotension were correlated with the SPX and the combined newborn and placenta weights. SPF measurements were unrelated to the maximum sensory block level, incidence of hypotension and ephedrine dose. We think that the SPX and the combined newborn and placenta weights have high predictive values for the spread of spinal anesthesia using hyperbaric bupivacaine and fentanyl. The SPX, with its ease of measurement from readily identifiable landmarks, may be useful in predicting the sensory block level and the incidence of hypotension after spinal anesthesia. To the best of our knowledge, this is the first study to investigate the predictive value of the distances between the symphysis pubis and the xiphoid process on sensory block levels following spinal anesthesia. Further research is needed in this regard.

**Acknowledgments**

The authors would like to thank Timur Köse and Banu Özgürel for their assistance with the statistical analyses in this study.
